# Bayesian binding and fusion models explain illusion and enhancement effects in audiovisual speech perception

**DOI:** 10.1371/journal.pone.0246986

**Published:** 2021-02-19

**Authors:** Alma Lindborg, Tobias S. Andersen

**Affiliations:** 1 Department of Psychology, University of Potsdam, Potsdam, Germany; 2 Section for Cognitive Systems, Department of Applied Mathematics and Computer Science, Technical University of Denmark, Kongens Lyngby, Denmark; Nottingham Trent University, UNITED KINGDOM

## Abstract

Speech is perceived with both the ears and the eyes. Adding congruent visual speech improves the perception of a faint auditory speech stimulus, whereas adding incongruent visual speech can alter the perception of the utterance. The latter phenomenon is the case of the McGurk illusion, where an auditory stimulus such as e.g. “ba” dubbed onto a visual stimulus such as “ga” produces the illusion of hearing “da”. Bayesian models of multisensory perception suggest that both the enhancement and the illusion case can be described as a two-step process of binding (informed by prior knowledge) and fusion (informed by the information reliability of each sensory cue). However, there is to date no study which has accounted for how they each contribute to audiovisual speech perception. In this study, we expose subjects to both congruent and incongruent audiovisual speech, manipulating the binding and the fusion stages simultaneously. This is done by varying both temporal offset (binding) and auditory and visual signal-to-noise ratio (fusion). We fit two Bayesian models to the behavioural data and show that they can both account for the enhancement effect in congruent audiovisual speech, as well as the McGurk illusion. This modelling approach allows us to disentangle the effects of binding and fusion on behavioural responses. Moreover, we find that these models have greater predictive power than a forced fusion model. This study provides a systematic and quantitative approach to measuring audiovisual integration in the perception of the McGurk illusion as well as congruent audiovisual speech, which we hope will inform future work on audiovisual speech perception.

## Introduction

When we see the face of a person speaking to us, our brains use both the auditory and visual input to understand what is being said. Seeing the speaker’s face enhances the perception of speech, especially in noisy conditions [1], and speeds up the neural processing of the speech signal [2]. A striking behavioural demonstration of audiovisual speech perception is the McGurk illusion, in which combining an auditory utterance (eg. “ba”) with incongruent visual speech (eg. “ga”) often causes a fused percept different from both the auditory and visual components (eg. “da”).

Since its discovery, the McGurk illusion has been studied extensively and several quantitative models have been developed to characterize this phenomenon as an optimal fusion of either discrete (eg. the Fuzzy Logical Model of Perception, [3]) or continuous (eg. maximum-likelihood estimation [4–6]) estimates of the auditory and visual cues. These “strong fusion” models hypothesise that the fused audiovisual percept is determined by the auditory and visual percepts weighted according to their reliability of information [3–5, 7]. That is, if one knows how the auditory and visual cues are perceived separately, one can directly predict the perception of them when combined. However, numerous studies have suggested that the information reliability principle may not sufficiently account for all the aspects of audiovisual speech perception. In fact, factors such as attention [8–10], audiovisual context [11], top-down expectations [12, 13], time offset between cues [14] and even spontaneous pre-stimulus brain activity [15] have all been shown to modulate the McGurk illusion. A strong fusion model cannot account for any of these effects.

Rather than a strong fusion process, audiovisual speech perception could perhaps then be better conceptualised as the brain performing Bayesian inference of the phonetical content of the audiovisual speech signal. When performing Bayesian inference, the brain estimates the *posterior* probability of a certain utterance being pronounced by combining a *prior* with the *likelihood* (i.e. the noisy representation of the auditory and visual speech cues). The *prior* captures the prior knowledge about the statistics of the world. In the multisensory case, this will be a prior which informs the brain’s belief about the probability of the cues originating from the same cause and thus whether they should be integrated. It could be informed by an early, low-level process of determining the correlation of auditory and visual inputs [16], as well as context cues and other more general prior knowledge.

A well-known Bayesian model of multisensory perception is Bayesian Causal Inference (BCI), according to which the brain in the binding stage computes the probability of the cues being caused by the same or separate events. Subsequently, a strong fusion model and a segregation model are combined by taking a weighted mean of their respective predictions, the strong fusion prediction weighted by the probability for a single cause and the segregation prediction weighted by the probability for separate causes [17–20]. Joint Prior models represent a slightly different Bayesian modelling paradigm, which instead of a discrete prior over the causal structure uses a continuous prior over the joint distribution of the auditory and visual features, determining to what extent they should be integrated [20–22]. Both of these Bayesian models reflect a multi-stage view of audiovisual speech perception [23–25], and could possibly be implemented in the brain as a fast route from visual motion areas to auditory cortex and a slower route through the STS for (iteratively) transmitting prediction errors [12, 26].

To our knowledge, no quantitative model of audiovisual speech perception has been tested in an experimental paradigm that manipulates both stages of audiovisual integration proposed by the Bayesian account, which we will from now on call the “binding” and “fusion” stage. Within the BCI literature the main experimental focus has for a long time been on the binding stage [17, 18, 20, 27], manipulating the assumption of common cause while keeping the strength of the cues constant. A few notable studies have successfully manipulated both the binding and fusion stages in spatial localization tasks [28–30], but this approach has not yet been tested in the audiovisual speech domain. In the strong fusion literature, on the other hand, the binding stage is omitted by assuming that all stimuli are maximally fused [3, 4, 31]. Clearly, the one-sided focus on either the binding or fusion step in previous audiovisual speech studies limits our understanding of what factors drive the perception of the McGurk illusion. A binding-focused paradigm would attribute a strong McGurk effect to a strong audiovisual binding, although it may also have been caused by difficulties in perceiving the auditory component of the signal, or by strong lipreading skills [32]. Conversely, in a strong fusion paradigm, subjects with low proportions of fusion responses would be assumed to have relatively high auditory precision, ignoring that the low degree of visual influence could be due to less audiovisual binding. Given the significant individual variability in susceptibility to the McGurk illusion [31–33], the respective effects of unisensory perception and multisensory binding should be clarified.

In this study, we apply the Joint Prior model [22, 34] and BCI to audiovisual speech perception. Furthermore, we evaluate the models in an experimental paradigm which targets both the binding and the fusion stage. We will manipulate the binding stage by introducing a temporal offset between the auditory and visual component. The McGurk illusion has been found to occur the most within a small interval of temporal offsets (30 ms audio lead to 170 ms audio lag), decreasing gradually with greater offsets [14]. Since the fusion stage depends on the relative precision of the auditory and visual cues, this stage of audiovisual integration will be experimentally manipulated by making either modality less reliable, i.e. by adding noise to the auditory or visual component of the stimulus. In order to correctly estimate the precision, each cue should be presented in a unimodal (audio-only or visual-only) condition, in addition to the audiovisual presentations.

Combining the approaches for manipulating the binding and fusion stages, respectively, our behavioral paradigm systematically manipulates the timing of the audiovisual stimuli as well as the auditory and visual signal to noise ratio (SNR), using both congruent and incongruent (McGurk) audiovisual stimuli. Our behavioural experiment is, to our knowledge, the first to vary both the effect of information reliability and differences in binding in audiovisual speech perception. This is necessary for testing the Bayesian models of audiovisual speech perception in full. Moreover, we will analyse to what extent the parameter values obtained from computational modelling can quantify the effects of binding and fusion on the behavioural responses.

### Model specification

In the Bayesian view, perception is achieved by combining the brain’s prior beliefs about the world (*prior*) with the incoming neural signal prompted by the stimulus (*likelihood*) to produce the probability for the neural signal being emitted by a specific stimulus (*posterior*). In line with previous *early fusion* models [4, 6, 18], we use a continuous internal representation for the auditory and visual cues, i.e. we assume that the brain estimates continuous-valued features of the spoken phonemes. These continuous unimodal estimates are combined via an integration rule to produce an audiovisual estimate, which is used to make the final phonetic judgment. In reality, phoneme identification is most probably a multidimensional problem but for computational feasibility we restrict our model to a one-dimensional representation of each modality (auditory and visual). In our study, since we will work with the consonants B, D and G, we can conceptualise the auditory and visual dimension as a continuous representation of the place of articulation, with B being articulated in the front of the mouth and G articulated furthest back.

**Fig 1:**
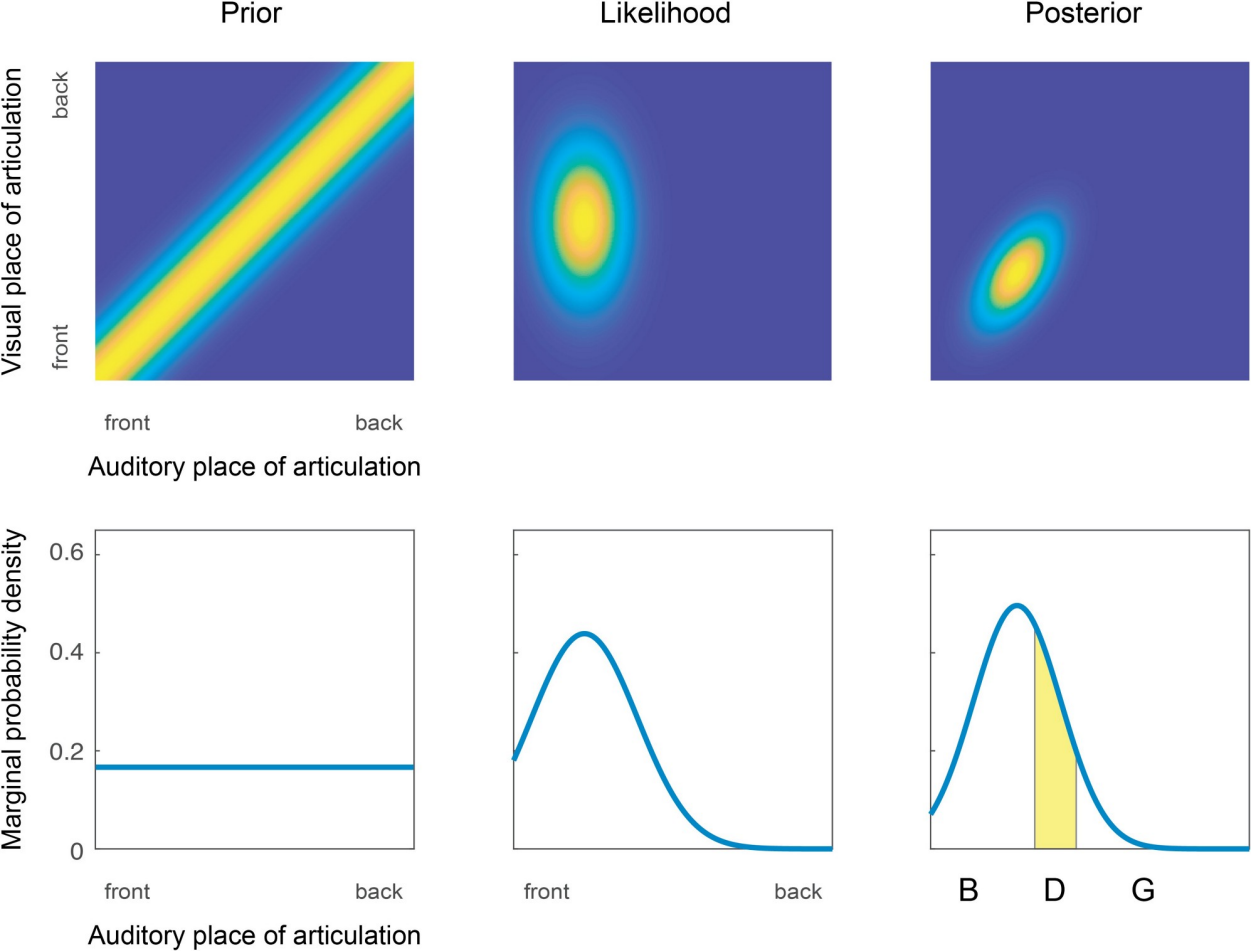
The Joint Prior model of audiovisual speech perception. Upper row: Example plots of prior, likelihood and posterior distributions. The horizontal axes represent the auditory dimension and the vertical dimension represents the visual dimension. The prior is a Gaussian ridge along the A = V diagonal, and the likelihood is a Gaussian (here depicted with greater variance in the visual dimension). The posterior distribution is also Gaussian, pulled in the direction of the A = V diagonal. Lower row: the marginal distribution of the prior, likelihood and posterior in the auditory dimension. Response boundaries (vertical lines) are applied to the posterior distribution and response probabilities are estimated as the probability mass (yellow area) delimited by the response boundaries.

The Joint Prior model of audiovisual speech perception builds on earlier work using two-dimensional priors for the joint distribution over two sensory dimensions [20–22, 34]. In this study, we use a Gaussian ridge along the A = V diagonal in the space of audiovisual speech features (see Fig 1) whose variance along the orthogonal diagonal, *σ*_*o*_^2^, determines the strength of audiovisual integration [20–22, 34]. The joint prior thus has one free parameter. Since this particular prior is specificed by its width from the diagonal, which also determines the strength of cross-modal binding, we will call it a *binding prior*. If there is a strong assumption of unity, *σ*_*o*_^2^ is small and posterior estimates will be forced towards the diagonal (i.e. the auditory and visual judgments will be the same; full integration). On the other hand, if there is a weak assumption of unity, *σ*_*o*_^2^ is large and posterior estimates will be close to the likelihood (little or no integration). The *likelihood* is a two-dimensionsal Gaussian describing the noisy representation of the incoming stimulus, with auditory and visual means (*μ*_*A*_, *μ*_*V*_) and precision parameters *r*_*A*_ = 1/*σ*_*A*_^2^ and *r*_*V*_ = 1/*σ*_*V*_^2^ (*σ*_*A*_^2^ and *σ*_*V*_^2^ being the variance of the Gaussian in the auditory and visual dimension, respectively). As in previous models [4, 5, 35] we assume conditional independence of the auditory and visual precision, so that, conditioned on the stimulus presented, the sensory noise will be independent in the two modalities and the likelihood thus has zero covariance.

By Bayes rule, the posterior is proportional to the product of the prior and the likelihood, which yields a Gaussian distributed posterior. Because participants are only required to report what they heard in the audiovisual condition, we marginalise the posterior over all possible visual representations to get the distribution of the auditory representations [17]. This is also a Gaussian distribution with mean

$$\mu_{AV}=w_{A}\mu_{A}+\left(1-w_{A}\right)\mu_{V}$$ (1)

and variance:

$$\sigma_{AV^{2}}=w_{A}\sigma_{A}{}^{2}$$ (2)

where *μ*_*A*_, *σ*_*V*_^2^, *μ*_*V*_, and *σ*_*V*_^2^ are auditory and visual mean and variance parameters and

$$w_{A}=\frac{2\sigma_{o}{}^{2}+\sigma_{V}{}^{2}}{2\sigma_{o}{}^{2}+\sigma_{V}{}^{2}+\sigma_{A}{}^{2}}$$ (3)

is the weight of the auditory mean in the combined estimate. Note that *w*_*A*_ will range between 0 (when the auditory precision is low) and 1 (when the visual precision is low). Thus, the posterior of the Joint Prior model incorporates the information reliability principle. However, we also see that the auditory weight is modulated by the prior variance. If the prior variance *σ*_*o*_^2^ is very large, the auditory weight will be close to 1 *regardless* of the auditory and visual precision because the auditory and visual signals are fully segregaged. On the other hand, as the prior variance approaches zero, the auditory weight will approach that of the strong fusion Maximum Likelihood Estimation (MLE) model [5].

In the BCI model, the posterior is a combination of two models–a strong fusion model and a full segregation model–weighted by the probability for common (P(C = 1)) and separate (P(C = 2)) causes, respectively [17, 18, 20]. Since the auditory and visual inputs must have either a common or separate causes and P(C = 1) and P(C = 2) hence sum to 1, the causal prior has one free parameter.

For both models, response probabilities are estimated by first restricting the representation space to a finite interval and then applying two response boundaries to divide the interval into three parts, corresponding to the three consonants (illustration in lower right corner of Fig 1). The response probability for a certain consonant is then calculated as the probability mass of the posterior that falls within its interval.

**Fig 2:**
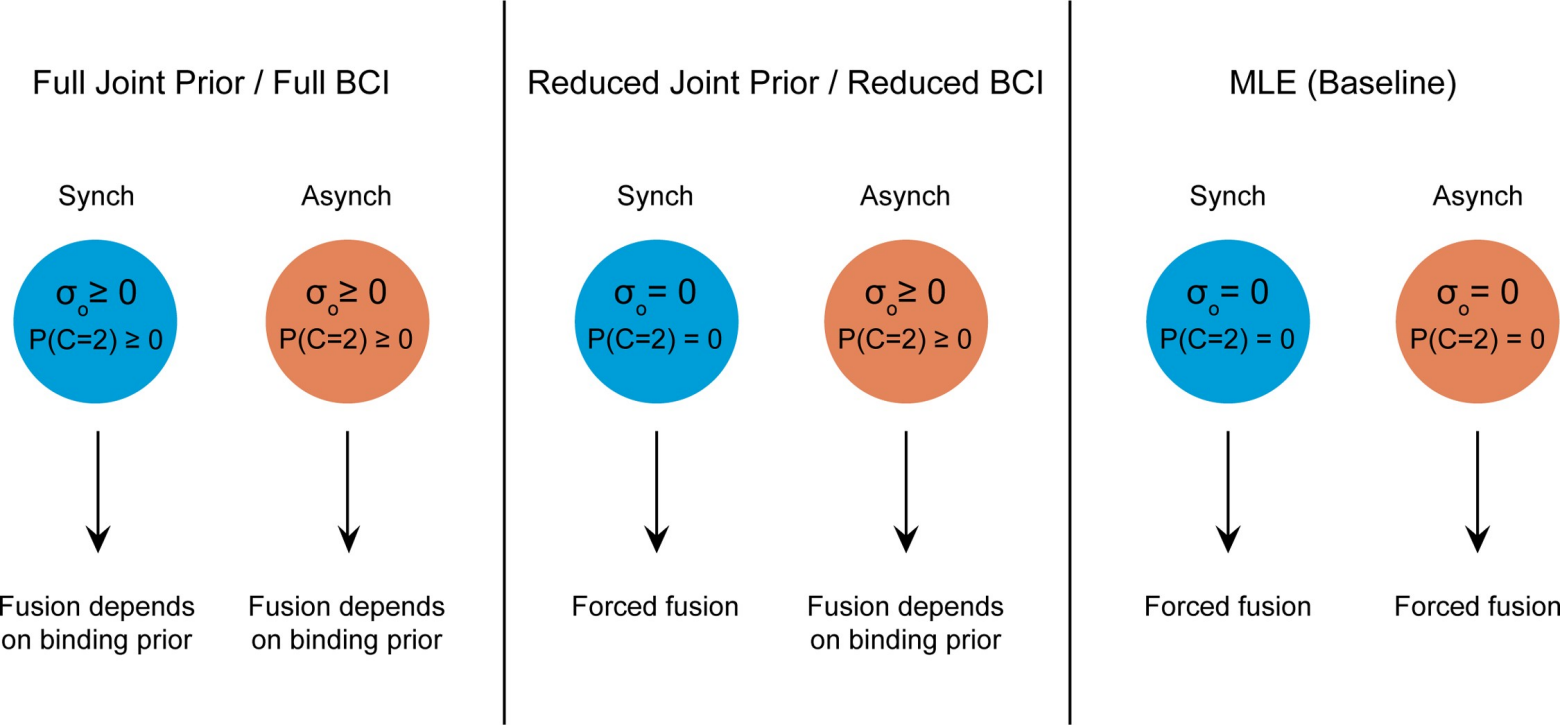
Prior structures. Illustration of the prior structure of each model compared in the study. A full derivation of the Joint Prior model of audiovisual speech perception is available in the supporting information.

In order to assess the importance of the prior on the predictive power of the models, we compare two different implementations of each model. In the Full implementation, we fit one prior parameter for each temporal offset condition. This entails fitting one prior variance parameter *σ*_*o*_^2^ for the synchronous stimuli and one for the asynchronous stimuli in the case of the Joint Prior model, and one P(C = 2) for each synchrony condition in the BCI. In the Reduced implementations we let the prior parameters vary freely in the asynchronous condition, but assume a strong fusion model in the synchronous condition, i.e. fixing the prior variance and P(C = 2) to zero in the Joint Prior and BCI, respectively. Finally, as a baseline for comparison we use the MLE model, which corresponds directly to either a Joint Prior model with a prior variance of zero in all conditions, or a BCI model with P(C = 2) set to zero in all conditions. See Fig 2 for illustration of the prior structure of the model implementations. An identical set of parameters pertaining to the model likelihood and response boundaries was fit in each model implementation: means for the perceptual categories B and G in the auditory and visual dimensions (4 parameters), auditory and visual variance parameters for each SNR level (6 parameters), and dividing the marginalised representational space into three categories (2 parameters). This means that the MLE had 12 free parameters in total, whereas the Reduced Bayesian models each had 13 free parameters and the Full Bayesian models had 14 free parameters.

## Methods

### Behavioural experiment

#### Participants

16 participants (10 female) with mean age 24.9 years (SD = 4.53) participated in the experiment in exchange for hourly payment. All subjects reported normal hearing and had normal or corrected-to-normal vision. Written consent was obtained from all participants and the experiment was approved by the Science-Ethics Committee for the Capital Region of Denmark (reference H-16036391).

#### Stimuli

Stimulus material was based on audiovisual recordings of a female native speaker of Danish uttering the syllables “Ba” and “Ga” (henceforth B and G). Videos were recorded at 25 frames/s and segments of 35 frames (1.45 s) were cut out using Adobe Premiere Pro software. In addition to congruent presentations of the recorded stimuli, McGurk fusion stimuli were created by combining visual G with auditory B, aligning the sound onset of the B with that of the (muted) G.

To manipulate the auditory and visual SNR, random noise was added to the stimuli. The noise was designed to match the spatiotemporal frequency content of the stimuli, as this type of noise is known to be efficient in masking both auditory [36, 37] and visual [38] stimuli. In the visual domain, noise was created by applying the Fourier transform to the three-dimensional (frame x horizontal position x vertical position) array of grayscale pixel values and replacing the phase angle values with uniformly distributed random numbers between 0 and 2π, while leaving magnitudes unchanged. Since the mouth area had more movement than the video on average and thus had a different frequency content, local noise was made for the mouth area to ensure that it could be sufficiently obscured. The noise was mixed with the signal by subtracting the mean pixel value and adjusting the contrast by scaling the pixel values of each video before adding them both to the mean pixel value. Three SNR levels (ranging from relatively clear to noisy) were created by varying the contrast settings for the signal and noise respectively. These contrast levels were found in pilot trials. An example image of the first author (AL) with applied visual noise corresponding to the SNR levels used in the experimental stimuli can be found in Fig 4B. Another speaker was used in the actual experimental stimuli.

In constructing the auditory noise, a short time Fourier transform was applied to each of the audio tracks and the magnitude spectrum of the onset of the respective consonants was computed. Subsequently, the mean of the two magnitude spectra was computed and combined with randomized phase angles in the same way as the visual case. This resulted in auditory noise matching the mean spectral profile of the two consonant onsets. The noise was faded in so that it reached its maximum at the earliest onset of the speech sound among all the stimuli (about 100 ms after start of video), and faded out at the end of the stimulus. The noise was then mixed with the speech sounds at relative dB 3, 9, and 15 in order to create stimuli with high, medium and low signal-to-noise ratio. These relative noise levels were determined in pilot trials.

Asynchronous versions of the stimuli were made by temporally shifting the audio to create a 500 ms audio lead. Although this stimulus onset asynchrony (SOA) is substantially larger than the “temporal window of integration” previously found [14, 25], we found in pilot trials that it was necessary to extend the window to 500 ms in order to produce a reliable effect of SOA. We believe that this extended time window of integration may be due to the decreased temporal resolution of our stimuli due to the added noise on both the auditory and visual stimulus components. Several studies have found that the time window of integration is not fixed, but depends on stimulus properties [19, 39–42]. Indeed, a study using synthetic audiovisual speech stimuli–and thus possibly lacking some high-frequency temporal information in both the auditory and visual domains–found that the SOA had to be extended to 533 ms to produce a significant decrease in audiovisual integration [43].

Audiovisual stimuli were created by varying the visual SNR by combining clear audio with clear, mid or noisy video and by varying the acoustic SNR by combining clear video with clear, mid or blurred audio. Auditory-only stimuli were displayed with a still image of the face, and in the visual only condition the videos were played without sound.

In order to make sure that subjects did not simply learn to discriminate between stimuli based on the patterns of the noise, 25 unique visual noise signals and 30 unique auditory noise signals were generated and combined with the stimuli such that any combination of the stimuli and noise signals appeared only once during the experiment.

#### Procedure

Participants were seated in a sound-isolated booth, where videos were presented on an LCD monitor and the audio was delivered at 65 dBA via an active loudspeaker placed below the screen. Videos were centred such that the mouth was displayed at the middle of the screen. The task was to identify the consonant uttered at each trial by choosing between the options “B”, “D” and “G”. A response screen was shown directly after each stimulus presentation, and responses were collected from the numeric keypad buttons “1”, “2” and “3” on the right-hand side of a standard computer keyboard. After the button press and a short delay of 200 ms, the next stimulus was presented. Subjects were instructed to focus on the mouth during the entirety of the experiment and to always report what they *heard*, except on visual-only trials in which they were instructed to lip read.

Stimuli were delivered in a pseudo-random order in blocks of 42 stimuli (one of each condition), ensuring that no stimulus would be presented more than twice consecutively. Each stimulus was presented 25 times, yielding 1050 presentations in total. The experiment took around 50 minutes to complete and contained a break halfway through.

### Model fitting & comparison

Models were fit by minimizing the negative log-likelihood of the data given the parameters, using the quasi-Newton optimization algorithm implemented in MATLAB’s fminunc function. Cross-validation over stimuli was applied during model fitting, meaning that all trials from one of the 42 experimental conditions were left out during model fitting in each cross-validation fold, and predictions were made on the left-out condition. The optimization was run 100 times with randomly selected initial points and the parameter set yielding the lowest negative log-likelihood was selected. Although the negative log-likelihood was used for model fitting, the cross-validation errors will be reported as root mean squared errors (RMSE). The reason for this is that the likelihood of left-out stimuli given the predicted response probabilities may sometimes be zero, and log(0) is not defined. This happens in the case where a response probability of zero or one is predicted and the observed response proportion is different from zero or one.

In order to keep model sensitivity in check, regularization was applied in the model fitting. Regularization penalises regions in the parameter space which yield unstable fits by putting priors on the parameter values. In the case of this experiment, very large estimates of auditory or visual precision (inverse variance) will cause such unstable fits by yielding highly peaked response probability distributions. With overly peaked probability distributions, a slight shift in category means or response boundaries will cause a big change in the predicted response probabilities, and consequently in the log-likelihood error. Thus, we penalise solutions with large auditory or visual precision by adding their sum scaled by a regularization constant λ to the negative log-likelihood function. This is equivalent to putting a Gaussian prior with zero mean and standard deviation 1/λ on the precision parameters. The value of λ = 7 was found by making a parameter sweep in the interval [10^−3^, 10^2^] (i.e. ranging from a very weak to a very strong prior) and selecting the value giving the lowest mean cross-validation RMSE on the left out stimuli over all three models.

## Results

### Behavioural results

**Fig 3:**
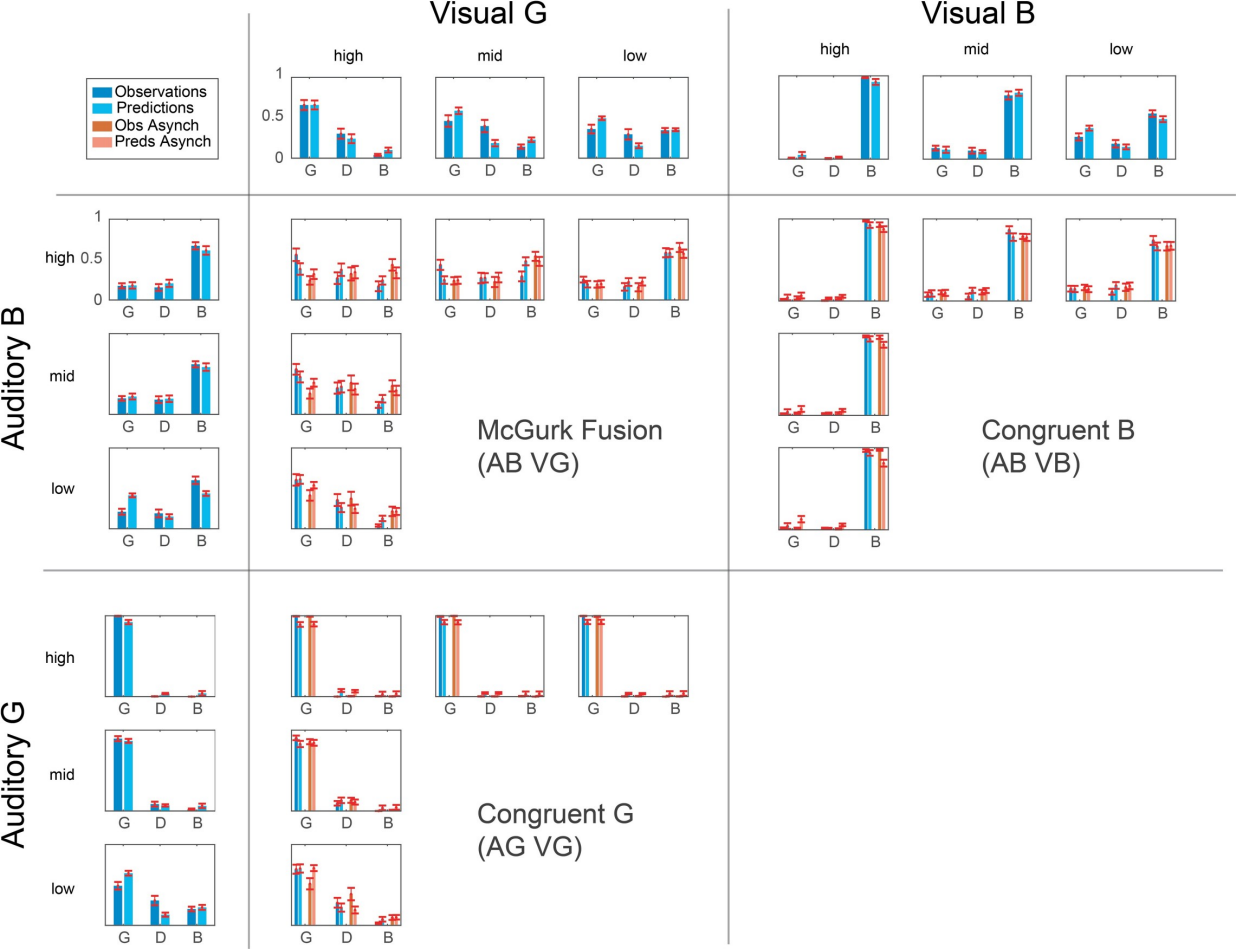
Behavioural responses and model predictions. Mean behavioural responses (dark bars) and model predictions (light bars) to visual-only (top row), auditory-only (left column) and audiovisual stimuli (central panels) for 16 participants. Error bars represent the standard error of the mean. Visual stimuli are divided into G (left compartment) and B (right compartment) and are presented with descending SNR (left: high SNR to right: low SNR within each compartment. Auditory stimuli are divided into B (top compartment) and G (bottom compartment) and are presented with descending SNR (top: high SNR to bottom: low SNR within each compartment). Each audiovisual stimulus is a combination of the auditory and aisual stimulus on the corresponding row and column, presented either in synchrony (blue bars) or out of sync (red bars). The model predictions displayed are cross-validation predictions from the Reduced Joint Prior model.

Fig 3 shows the mean behavioural responses (dark bars) to auditory-only, visual-only and audiovisual stimuli. The behavioural data are available in full in the supporting information.

For the unimodal (auditory-only and visual only) stimuli, we expected that accuracy would decrease when more noisy stimuli are presented. We tested this hypothesis separately for auditory-only and visual-only stimuli using generalized mixed-effects models (GLMs). The dependent variable was the number of correct responses and because this is a binomial count (correct vs. incorrect), a binomial GLM with a logit link function was used. The fixed effects were Stimulus (B or G) and SNR (high, mid or low) and Subject was a random effect. For auditory-only stimuli, an Analysis of Deviance (Type II Wald chi-squared tests) revealed main effects of Stimulus (p < 0.001) and SNR (p < 10^−14^), as well as a Stimulus x SNR interaction (p < 10^−15^). Follow-up non-parametric pairwise tests revealed that the main effect of SNR and the Stimulus x SNR interaction reflected a decrease in correct responses with lower SNR for G (p < 0.001 for low vs. mid and mid vs. high SNR, one-sided Wilcoxon signed-rank tests), whereas for B the only significant difference was between low and high SNR (p. < 0.0003).

For the visual-only stimuli we used the same GLM approach as the auditory-only stimuli, revealing main effects of Stimulus (p < 10^−15^) and SNR (p < 10^−15^) and a SNR x Stimulus interaction (p < 10^−15^). Follow-up one-sided tests of high vs. mid SNR and mid vs. low SNR showed that lower SNR led to fewer correct responses for both B and G (p < 0.01 in all pairwise Wilcoxon signed-rank tests). This confirms our hypothesis that accuracy decreased when more noise was added. The additional main effect of Stimulus and the SNR x Stimulus interaction possibly reflect that the responses to visually more salient B were more correct and more affected by SNR than the responses to G.

To summarise, responses to both auditory-only and visual-only stimuli support the hypothesis that SNR modulates behavioural responses. We additionally found a main effect of Stimulus and Stimulus x SNR interaction effects in opposing directions for the two modalities: whereas responses to auditory G were in general more correct and more affected by SNR than those to auditory B, the converse was true for the visual stimuli where responses were more correct and more strongly modulated by SNR for B than G.

For McGurk stimuli, we have two hypotheses regarding the behavioural responses:

1. The responses corresponding to the auditory component of the stimuli (i.e. B; henceforth “auditory responses”) will decrease compared to the auditory-only condition due to the addition of an incongruent visual stimulus.
2. That responses will be modulated by SOA and SNR in the following manner: The auditory responses should be larger for asynchronous stimuli compared to synchronous because the SOA will disrupt the illusion, and low auditory SNR should lead to fewer auditory responses wheras low visual SNR should lead to more auditory responses.

In testing Hypothesis 1, we compared responses to the clear auditory B to the same stimulus presented in synchrony with a clear visual G, revealing a significant decrease in correct responses in the audiovisual condition (p < 0.0003, one-sided Wilcoxon signed-rank test). This effect was also found when the visual stimulus was weaker (p < 0.01 for mid and low Visual SNR, one-sided Wilcoxon signed-rank tests). In testing Hypothesis 2, we fit a GLM with fixed effects of SOA (synchronous or asynchronous), Auditory SNR and Visual SNR (high, mid and low, respectively). An Analysis of Deviance (Type II Wald chi-squared tests) revealed significant main effects of SOA, Auditory SNR and Visual SNR (all p < 10^−6^) and an interaction effect of SOA x Visual SNR (p < 10^−7^). In order to test whether the direction of these effects was in line with Hypothesis 2, we used one-sided pairwise Wilcoxon signed-rank tests. The effect of SOA reflected that asynchronous stimuli yielded more auditory responses than synchronous stimuli (p < 0.02 in all pairwise tests of the five fusion stimuli). Consistent with our hypothesis on how responses are modulated by the relative precision of the auditory cues, the main effect of Auditory SNR was due to the fact that auditory responses decreased for the lower auditory SNR levels (p < 0.05 in all pairwise one-sided tests of low SNR vs. mid SNR and mid SNR vs. high SNR). Conversely, the main effect of Visual SNR reflected an increase in auditory responses for the lower visual SNR levels (p < 0.005 in all pairwise tests of high SNR < mid SNR and mid SNR < low SNR). Thus, our data support both of our hypotheses regarding the McGurk stimuli. Additionally, we found a SOA x Visual SNR interaction reflecting that synchronous and asynchronous stimuli were differently affected by manipulation of the visual SNR.

For Audiovisual congruent stimuli, we hypothesise that 1) subjects will be more correct in perceiving the stimuli compared to the audio-only condition and 2) that this enhancement effect will be modulated by Auditory and Visual SNR as well as SOA, as adding noise to either component or adding asynchrony should decrease the number of correct responses. Testing Hypothesis 1, we found a significant increase in correct responses when adding a synchronous presentation of a clear visual B to a clear auditory B (p < 0.0003, Wilcoxon signed-rank test), as well as with less clear visual B (p < 0.0002 and p = 0.0245 for mid and low visual SNR, respectively). For Audiovisual G compared to auditory-only G, there was no improvement in the high and mid SNR conditions, possibly because identification was at ceiling level in the auditory-only condition. In the low Auditory SNR condition, however, there was an improvement in identifying G when a synchronous visual stimulus was added (p < 0.0005, Wilcoxon signed-rank test). Testing Hypothesis 2, we again fit a mixed-effects GLM to the binomial count of correct vs. incorrect responses, using a logit link function. The fixed effects were Stimulus (B or G), SOA (synchronous or asynchronous), Auditory SNR (high, mid, low) and Visual SNR (high, mid, low); and Subject was a random effect. An Analysis of Deviance (Type II Wald chi-square tests) revealed significant main effects of Stimulus, SOA, Auditory SNR and Visual SNR (all p < 10^−6^) and interaction effects of Stimulus x Auditory SNR (p < 10^−6^) and Stimulus x Visual SNR (p = 0.0012). In order to uncover whether the main effects of SNR and SOA were in the hypothesised direction, we again conducted one-sided pairwise Wilcoxon signed-rank tests. When testing whether the SOA effect was in the hypothesised direction, we found a decrease in correct responses for asynchronous presentations for auditorily mid and noisy presentations of G as well as all auditorily clear presentations of B (p < 0.03). However, for auditorily clear G and auditorily mid and noisy B there was no effect of SOA, possibly due to ceiling effects. The main effect of Auditory SNR reflected a significant decrease in correct responses to G as the auditory SNR decreased (p < 0.001 for high vs. mid and mid vs. low in both SOA conditions), whereas there was no such effect for B. Conversely, the main effect of Visual SNR was accompanied by a significant decrease in correct reseponses to B as the visual SNR decreased (p < 0.001 for high vs. mid and mid vs. low in synchronous and asynchronous conditions), whereas no significant effect was found for G. These main effects are in line with Hypothesis 2. The additional main effect of Stimulus possibly reflects differences in unisensory perception. Our data thus support both of our hypotheses regarding the congruent Audiovisual stimuli. Additionally, we found interaction effects which were not part of our hypotheses, which will be interpreted in the Discussion.

### Modelling

**Fig 4:**
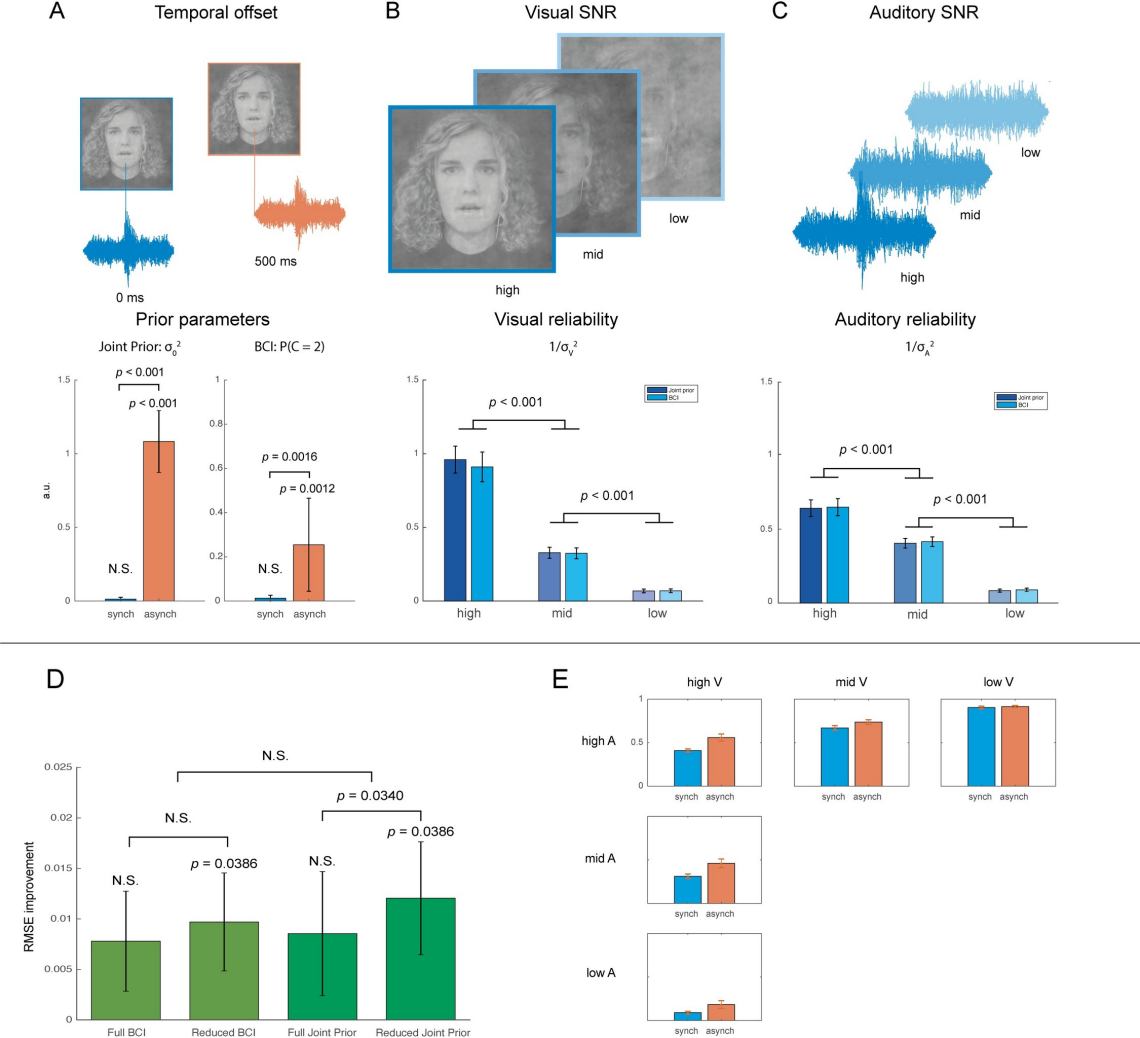
Modelling results. **A)** Prior parameters for synchronous and asynchronous stimuli: binding parameter (0 = full binding, infinite = no binding) for the Full Joint Prior model, and probability of separate causes (0 = full binding, 1 = no binding) for the Full BCI model. **B)** Auditory and **C)** visual precision parameters of the Reduced Joint Prior and BCI for clear to noisy stimuli (left to right). The images depict the first author. Error bars represent SEM. **D)** Improvement in test error over baseline (the Maximum likelihood model) for the Reduced and Full Bayesian model implementations. Error bars represent SEM. **E)** Auditory weight in the Reduced Joint Prior model, plotted by SNR and SOA.

Fig 4D shows the test error (RMSE) improvement of the Bayesian model implementations compared to the MLE model, here used as a baseline for comparison. The Reduced BCI and Reduced Joint Prior both had lower cross-validation error than the MLE model (p < 0.04 in both pairwise Wilcoxon signed-rank tests). The Reduced Joint Prior model had lower cross-validation error than the full implementation (p = 0.0340), but for BCI there was no significant difference between the full and reduced implementations. Moreover, pairwise tests revealed a significant difference between Reduced BCI and MLE (p = 0.0386), but no significant differences between the Reduced BCI and the Full BCI. Finally, there was no significant difference between MLE and Full Joint Prior or Full BCI, nor between the Joint Prior and BCI in either the Full or Reduced implementation. Thus, model comparison clearly favours the Reduced Bayesian implementations.

#### Model predictions

Fig 3 shows the cross-validation predictions of the Reduced Joint Prior model (i.e. the prediction for each condition in the cross-validation fold where it was left out) together with the behavioural data. The figure indicates that the model predictions generally follow the patterns in the data for McGurk Fusion stimuli as well as for congruent G and B. There are two notable exceptions to the good prediction performance. First, in the noisy auditory-only condition the model substantially underestimates accuracy for B and overestimates accuracy for G. Second, in the noisy visual-only condition the model over-estimates the accuracy for G and under-estimates the accuracy for B.

#### Model parameters

Reviewing the estimated prior parameters, we use the Full implementations of the Bayesian models for illustration in Fig 4A (the Reduced implementations had their prior parameters fixed to zero in the synchronous condition). We see that for synchronous stimulus presentations, the prior parameters indicate close to maximal binding: neither the prior variance in the Joint Prior model nor the probability of separate causes in the BCI is significantly different from zero. This provides further support for the Reduced model implementations. In the asynchronous condition, however, binding was not maximal, as reflected in both the prior variance of the Joint Prior model and the probability of separate causes in the BCI being significantly greater than zero (one-sided t-tests yielding p < 0.0001 and p = 0.0012 respectively).

Reviewing the likelihood parameters, Fig 4B and 4C shows the estimated auditory and visual precision parameters for the Reduced Joint Prior model, for each SNR level. We tested our hypothesis that the value of the precision parameters decrease with decreasing SNR in the Joint Prior and BCI models separately. One-sided Wilcoxon signed-rank tests revealed that this was indeed the case. The precision in the clear auditory condition was significantly higher than that of the middle condition (p < 0.001 for each model), which in turn was higher than the noisy condition (p < 0.001 for each model). The visual parameters followed the same pattern, where clear visual had higher precision than the middle condition (p < 0.001 for each model), which had higher precision than the noisy condition (p < 0.001 for each model). Moreover, we see that the highest sensory reliability is that of the clear visual condition, whereas for the other SNR levels the auditory and visual reliabilities are more similar to one another.

#### Weighting of cues depend on relative precision and binding

A feature of the Joint Prior model is that the posterior distribution is a Gaussian with a mean that is a weighted combination of the means for the unisensory response distributions (see Eq 3). Thus, having fit the model to our behavioural data, we can explicitly estimate the relative influence of the auditory and visual modalities. Fig 4E shows the auditory weights as a function of audiovisual stimulus and SOA. The weight given to the auditory modality varies with the relative auditory precision of the stimulus, growing incrementally from the lowest relative auditory precision (low auditory SNR, high visual SNR) to the highest relative auditory precision condition (high auditory SNR, low visual SNR). We conducted one-sided Wilcoxon signed-rank tests of each pair of adjacent conditions (i.e., low auditory SNR should have a smaller auditory weight than mid auditory SNR, etc), revealing a significant difference for all pairs of both synchronous and asynchronous stimuli (all p < 0.01).

Moreover, the auditory weight is consistently higher for synchronous compared to asynchronous presentations, reflecting the decreased binding in the asynchronous condition. Indeed, a mixed-effects ANOVA (fixed effects: Auditory SNR, Visual SNR, SOA, random effect: Subject) on the auditory weights revealed a significant effect of Auditory SNR (p < 10^−10^), Visual SNR (p < 10^−10^) and SOA (p < 10^−9^), as well as a Visual SNR x SOA interaction (p = 0.001), because the difference between synchronous and asynchronous weights were smaller for noisy visual stimuli.

## Discussion

### Bayesian models can predict audiovisual enhancement and illusion effects

Overall, the behavioural responses to audiovisual stimuli was in line with the information reliability principle: responses were modulated by both auditory and visual SNR. Higher visual SNR led to more visual influence, whereas higher auditory SNR led to less visual influence. Thus, our data clearly illustrate that auditory and visual speech cues are weighted according to their relative reliabilities. Moreover, the weaker visual influence in the asynchronous conditions shows that the temporal offset affects the extent to which the brain integrates the cues. Additionally, there was a significant SOA x Visual SNR interaction, which although not part of our initial hypothesis is in line with a binding and fusion model: since a low SNR visual stimulus will have a negligible influence on perception, we would not expect an effect of temporally offsetting these stimuli. Thus, our experimental paradigm successfully taps in to both the prior and likelihood stages of the Bayesian models, corresponding to the binding and fusion stages of the two-stage models of audiovisual integration [12, 18, 23, 26].

The responses to congruent audiovisual stimuli follow the same pattern as the McGurk responses: information reliability as well as SOA modulate these responses in a manner consistent with the predictions of the Bayesian models. However, some differences between B and G are worth noticing. Whereas the subjects were near-perfect in perceiving the G stimuli regardless of visual noise (probably due to a ceiling effect), the B responses were clearly affected by visual SNR. On the other hand, the number of correct responses to G decreased when auditory noise was added, whereas the B responses were unaffected by auditory SNR. Considering the physical properties of the stimuli, this pattern does not come as a surprise. The hard *G* in “ga” contains a velar burst which is relatively easy to perceive even when masked with noise [44], whereas the visual “ga” is harder to distinguish from “da”. Conversely, the visual “ba” starts with a complete closing of the lips and is hence very salient, whereas the auditory “ba” is harder to perceive. Thus, the observed pattern could be explained by differences in information reliability between congruent B and G. The difference between B and G is also reflected in the unimodal responses: visual-only B was better perceived and responses were more strongly modulated by SNR than for visual G, whereas auditory-only G was better perceived and showed a stronger SNR modulation than auditory B. Auditory-only B does not seem to be modulated by SNR at all, which calls into question whether our noise masking was in fact efficient for this consonant. However, there was a clear auditory SNR modulation when the same B consonant was presented in the McGurk condition, and thus it seems that the precision of the auditory cue was in fact different in the different SNR levels. Hence, we believe that the apparent absence of SNR effect in the auditory-only B condition may possibly be due to subjects learning to discriminate the small number of distinct auditory-only stimuli presented during the experiment.

Regardless of the differences in information reliability between B and G, we have presented a model that uses the same set of auditory and visual precision parameters for both consonants. This model design may be the cause behind the consistent prediction errors for the noisy auditory-only (where the model overestimates the precision of B and underestimates that of G) and visual-only (where B has too low precision and G has too high precision) conditions. This raises the question of why a separate set of precision parameters for B and G was not used. We did in fact implement such a model but it proved to be overly flexible, as reflected in a substantially higher generalization error than the models presented in this study.

The fact that the Reduced Joint Prior and Reduced BCI models had significantly lower test error than the MLE model shows that the unisensory signals alone are not sufficient for predicting behaviour in the asynchronous condition. Here, audiovisual integration is not maximal as assumed by the MLE model. Rather, the strength of integration varies with the prior binding parameter, which statistically differed from zero (maximal binding) in our data.

On the other hand, releasing the maximal fusion assumption for the synchronous condition did *not* increase prediction accuracy. The Full Joint Prior and Full BCI implementations did not yield a lower cross-validation error than their reduced counterparts; on the contrary, they were significantly less accurate. This may seem counter-intuitive, as the reduced models span a subspace of the solutions of their full counterparts. However, the higher cross-validation error of the Full Joint Prior and Full BCI models means that they are overly flexible to the data and hence do not generalise as well to unseen data as their reduced counterparts. Thus, our results suggest that a strong fusion model may be sufficient for predicting responses to temporally aligned audiovisual speech, whereas a free binding parameter is needed to predict the perception of out-of-sync speech.

### Relationship between the Joint Prior and BCI models

The Bayesian Causal Inference (BCI) model is the most commonly used Bayesian model of perception in recent literature on multisensory perception [17–20], whereas the Joint Prior was more widely used in earlier work [20–22]. As we have described, the Joint Prior defines a continuous prior parametrised by its variance across the A = V diagonal, whereas the BCI models the prior as the probability for the auditory and visual cues to have a common cause. The posterior distribution of the BCI is then a combination of a full integration (common cause, corresponding to a Joint Prior model with zero prior variance) and full segregation (separate causes, corresponding to Joint Prior with infinite prior variance) estimate, weighted by their respective probabilities. This causes the posterior distribution to be bimodal, as we now have a sum of two Gaussian distributions. Since the perception of our stimuli fall into three categories–corresponding to three intervals in the representational space delimited by response boundaries–we cannot judge whether the posterior distribution is unimodal or bimodal. Indeed, our model comparison revealed no significant differences between the Joint Prior and BCI models. Which of these models more accurately describes the brain’s processing of audiovisual speech stimuli remains an open question. However, they each allow us to take a certain viewpoint on real-world data. Whereas BCI estimates the probability of a common cause (as illustrated in Fig 4A), the Joint Prior model instead estimates the relative contribution of the auditory and visual modalities. This gives us a measure of how much the prior and likelihood contribute to the final estimate (as illustrated in Fig 4E).

### Quantifying audiovisual integration of speech

In the audiovisual speech perception literature there is a long-standing issue of how to quantify audiovisual integration [45]. Although the McGurk illusion rate has often been used as a behavioural marker for audiovisual integration, the absence of unisensory conditions have often made it unclear how much of the illusion rates can be attributed to unisensory performance and how much is due to actual binding or integration. Moreover, the fact that it relies on an incongruent, non-naturalistic stimulus has made the validity of the McGurk illusion as a measure of audiovisual integration disputed [46]. Our study suggests a remedy to these problems by combining a more complete behavioural paradigm with computational modelling. The inclusion of unisensory conditions allows for estimation of the separate contributions of multisensory binding and reliability weighting to the final response. Additionally, our experimental paradigm generalizes from the McGurk illusion to the enhancement effect for congruent audiovisual stimuli, using the same parameters for predicting both of these behavioural effects. This suggests a paradigm for future electrophysiology and imaging studies aiming to measure audiovisual integration of speech in a systematic manner.

## Conclusion

We have evaluated two Bayesian models of audiovisual speech perception–the Joint Prior model and BCI–in a behavioural paradigm which systematically manipulates both the binding prior and the relative weighting of cues. Although there are multiple studies using Bayesian modelling for multisensory behavioural paradigms, ours is (to our knowledge) the first audiovisual speech experiment which manipulates stimulus properties attributed to both the prior and the likelihood. Fitting a full-scale and a reduced version of our models and comparing it to a maximum-likelihood model with a fixed prior we found that the reduced model, which assumes maximum integration for synchronous stimuli and allows the binding prior to vary for asynchronous stimuli, performed best in predicting unseen data. Thus, in standard experimental paradigms with synchronous presentations it may well be that a maximum-likelihood model is sufficient to predict behavioural responses, if the likelihood parameters are allowed to vary between subjects. This is in line with findings that maximum-likelihood models, in standard experimental paradigms with synchronous presentations, predicts behavioural responses well if the likelihood parameters are allowed to vary between subjects [4, 6].

## Supporting information

S1 File Derivation. Derivation of the Joint Prior model of audiovisual speech perception.
(PDF)

S2 File Behavioural data. (ZIP)
