# Supplementary material for: Bayesian binding and fusion models explain illusion and enhancement effects in audiovisual speech perception
Source: PLoS One. 2021 Feb 19;16(2):e0246986. doi: 10.1371/journal.pone.0246986 (PMC7895372; doi:10.1371/journal.pone.0246986)
Supplement: S1 File — Derivation of the Joint Prior model of audiovisual speech perception. (PDF) [file pone.0246986.s001.pdf]

1 Prior

Let the stimulus $\mathbf{S}$ have a 2D Gaussian prior distribution with zero mean and variance parameters specifying the variance along the diagonal along the line $x = y$, (σ_d^2) and orthogonal to that diagonal (σ_o^2). By a 45 degree clockwise rotation of a normal distribution with the diagonal covariance matrix

$$\mathbf{\Lambda} = \begin{bmatrix} \sigma_d^2 & 0 \\ 0 & \sigma_o^2 \end{bmatrix} \tag{1}$$

we can parametrize our prior by using the rotation matrix

$$\mathbf{U} = \frac{1}{\sqrt{2}} \begin{bmatrix} 1 & -1 \\ 1 & 1 \end{bmatrix}, \tag{2}$$

and the relation

$$\mathbf{X} \sim \mathbf{U} \cdot \mathcal{N}(0, \mathbf{\Lambda}) \Leftrightarrow \mathbf{X} \sim \mathcal{N}(0, \mathbf{U\Lambda U}^{\mathrm{T}}). \tag{3}$$

(ref. Wkipedia.) This yields a Gaussian with mean $\mu_p = \mathbf{0}$ and

$$\mathbf{\Sigma_p} = \mathbf{U\Lambda U}^{\mathrm{T}} = \frac{1}{2} \begin{bmatrix} \sigma_o^2 + \sigma_d^2 & \sigma_d^2 - \sigma_o^2 \\ \sigma_d^2 - \sigma_o^2 & \sigma_o^2 + \sigma_d^2 \end{bmatrix}. \tag{4}$$

2 Likelihood

Let the likelihood be a 2D Gaussian with

$$\mu_l = \begin{bmatrix} \mu_A \\ \mu_V \end{bmatrix}, \ \mathbf{\Sigma}_l = \begin{bmatrix} \sigma_A^2 & 0 \\ 0 & \sigma_V^2 \end{bmatrix} \tag{5}$$

3 Posterior

The posterior distribution follows from the result that the product of two multivariate Gaussian densities is itself a multivariate Gaussian, scaled by a constant, i.e.

$$\mathcal{N}(\mu_p, \mathbf{\Sigma_p}) \cdot \mathcal{N}(\mu_l, \mathbf{\Sigma}_l) = c \cdot \mathcal{N}(\mu^*, \mathbf{\Sigma}^*). \tag{6}$$

Furthermore, it can be shown that

$$\mathbf{\Sigma}^* = (\mathbf{\Sigma_p}^{-1} + \mathbf{\Sigma}_l^{-1})^{-1} \tag{7a}$$

$$\mu^* = \mathbf{\Sigma}^*(\mathbf{\Sigma_p}^{-1}\mu_p + \mathbf{\Sigma}_l^{-1}\mu_l) = \mathbf{\Sigma}^*(\mathbf{\Sigma_p}^{-1} \cdot \mathbf{0} + \mathbf{\Sigma}_l^{-1}\mu_l) = \mathbf{\Sigma}^* \cdot \mathbf{\Sigma}_l^{-1}\mu_l. \tag{7b}$$

By Bayes theorem we have

$$P(B|A) = \frac{P(A|B)P(B)}{P(A)} \tag{8}$$

and since we know that the posterior distribution integrates to 1 (as does a multivariate normal distribution), we can conclude that $c = P(A)$ and drop that term from our further calculations.

Inserting our parametrizetions of the prior and likelihood covariance matrices into Equation (7a), we get

$$\mathbf{\Sigma}^* = a \cdot \begin{bmatrix} \sigma_A^2(2\sigma_d^2\sigma_o^2 + \sigma_d^2\sigma_V^2 + \sigma_o^2\sigma_V^2) & \sigma_A^2\sigma_V^2(\sigma_d^2 - \sigma_o^2) \\ \sigma_A^2\sigma_V^2(\sigma_d^2 - \sigma_o^2) & \sigma_V^2(\sigma_A^2\sigma_d^2 + \sigma_A^2\sigma_o^2 + 2\sigma_d^2\sigma_o^2) \end{bmatrix} \tag{9}$$

where

$$a = \frac{1}{\sigma_A^2\sigma_d^2 + \sigma_A^2\sigma_o^2 + 2\sigma_d^2\sigma_o^2 + 2\sigma_A^2\sigma_V^2 + \sigma_d^2\sigma_V^2 + \sigma_o^2\sigma_V^2}. \tag{10}$$

Similarly, we have

$$\mu^* = a \cdot \begin{bmatrix} \mu_A(2\sigma_d^2\sigma_o^2 + \sigma_d^2\sigma_V^2 + \sigma_o^2\sigma_V^2) + \mu_V\sigma_A^2(\sigma_d^2 - \sigma_o^2) \\ \mu_V(\sigma_A^2\sigma_d^2 + \sigma_A^2\sigma_o^2 + 2\sigma_d^2\sigma_o^2) + \mu_A\sigma_V^2(\sigma_d^2 - \sigma_o^2) \end{bmatrix}. \tag{11}$$

Now, we let the variance on the diagonal go to infinity, effectively shaping the prior into a ridge along the diagonal. This yields

$$\tilde{\mathbf{\Sigma}} = \lim_{\sigma_d^2 \to \infty} \mathbf{\Sigma}^* = b \cdot \begin{bmatrix} \sigma_A^2(2\sigma_o^2 + \sigma_V^2) & \sigma_A^2\sigma_V^2 \\ \sigma_A^2\sigma_V^2 & \sigma_V^2(\sigma_A^2 + 2\sigma_o^2) \end{bmatrix} \tag{12a}$$

$$\tilde{\mu} = \lim_{\sigma_d^2 \to \infty} \mu^* = b \cdot \begin{bmatrix} \mu_V\sigma_A^2 + \mu_A(2\sigma_o^2 + \sigma_V^2) \\ \mu_A\sigma_V^2 + \mu_V(\sigma_A^2 + 2\sigma_o^2), \end{bmatrix} \tag{12b}$$

where

$$b = \frac{1}{2\sigma_o^2 + \sigma_A^2 + \sigma_V^2}. \tag{13}$$

Since we are only interested in the auditory responses, we find the marginal posterior distribution with respect to the auditory response, which corresponds to the first dimension in our model.

As the marginal distribution of a 2D Gaussian is a univariate Gaussian with mean and variance corresponding to the mean and covariance of that dimension in the bivariate distribution, we get

$$\tilde{\mu}_A = \frac{\mu_V \sigma_A^2 + \mu_A(2\sigma_o^2 + \sigma_V^2)}{2\sigma_o^2 + \sigma_A^2 + \sigma_V^2} = \frac{\sigma_A^2}{2\sigma_o^2 + \sigma_A^2 + \sigma_V^2}\mu_V + \frac{2\sigma_o^2 + \sigma_V^2}{2\sigma_o^2 + \sigma_A^2 + \sigma_V^2}\mu_A \tag{14a}$$

$$\tilde{\sigma}_A^2 = \frac{2\sigma_o^2 + \sigma_V^2}{2\sigma_o^2 + \sigma_A^2 + \sigma_V^2}\sigma_A^2 \tag{14b}$$

Thus, $\tilde{\mu}_A$ is a weighted mean of μ_A and μ_V with non-negative weights summing to 1 and depending on the relative variance of the respective dimensions (prior variance and auditory and visual variance in the likelihood). Similarly, $\tilde{\sigma}_A^2$ is a scaling of σ_A^2 by the same scaling constant as that applied to μ_A in equation (14a), and since that scaling constant lies on the interval $[0, 1]$ we can conclude that $0 \leq \tilde{\sigma}_A^2 \leq \sigma_A^2$.

In the strong integration case, we would have $\sigma_o^2 = 0$; that is, a delta spike along the main diagonal. In that case, equations (14a) and (14b) reduce to (respectively)

$$\tilde{\mu}_A = \frac{\sigma_A^2}{\sigma_A^2 + \sigma_V^2}\mu_V + \frac{\sigma_V^2}{\sigma_A^2 + \sigma_V^2}\mu_A = \frac{\frac{1}{\sigma_V^2}}{\frac{1}{\sigma_A^2} + \frac{1}{\sigma_V^2}}\mu_V + \frac{\frac{1}{\sigma_A^2}}{\frac{1}{\sigma_A^2} + \frac{1}{\sigma_V^2}}\mu_A, \tag{15a}$$

$$\tilde{\sigma}_A^2 = \frac{\sigma_A^2 \sigma_V^2}{\sigma_A^2 + \sigma_V^2} \tag{15b}$$

which is exactly the maximum likelihood estimate as described by Ernst & Banks (2002), and Andersen (2015).

On the other hand, if we let $\sigma_o^2 \to \infty$ we get a flat prior, which should yield no integration of the auditory and visual cues (because there is no prior assumption that the cues are related). It is easy to see that our model fulfills this property, since

$$\lim_{\sigma_o^2 \to \infty} \tilde{\mu}_A = \mu_A \tag{16}$$

$$\lim_{\sigma_o^2 \to \infty} \tilde{\sigma}_A^2 = \sigma_A^2 \tag{17}$$

In the intermediate cases where σ_o^2 is nonzero and finite, it will shift $\tilde{\mu}_A$ towards μ_A compared to the maximum likelihood model, with a small

shifts for small σ_o^2 and bigger shifts for big σ_o^2. This corresponds intuitively to the notion that a narrow joint prior along the diagonal yields a stronger integration (i.e. higher visual influence on the auditory percept), whereas a more flat prior corresponds to a weaker assumption of a common cause of the cues and thus weights the visual information lower in computing the posterior auditory representation. Similarly, large σ_o^2 will shift $\tilde{\sigma}_A^2$ towards σ_A^2 whereas small values will shift it towards the maximum likelihood estimate.
